# Spouses’ personalities and marital satisfaction in Chinese families

**DOI:** 10.3389/fpsyg.2025.1480570

**Published:** 2025-02-05

**Authors:** Li Jia, Gerrit Antonides, Zhuomin Liu

**Affiliations:** ^1^School of Economics, Sichuan University, Chengdu, China; ^2^Department of Social Sciences, Wageningen University, Wageningen, Netherlands

**Keywords:** marital satisfaction, happiness, personality, actor effects, partner effects

## Abstract

**Introduction:**

Considering the traditional Confucian values prevalent in Chinese society, we study the effects of the spouses’ personalities and household sociodemographic circumstances on the marital satisfaction of marriage partners.

**Methods:**

We evaluate the factors that contribute to marital satisfaction, using the 2018 wave of the Chinese Family Panel Survey, including 6,603 households. We use information on both spouses’ Big-Five personality traits, and marital satisfaction. In addition, the effects of the household’s sociodemographic factors on the spouses’ marital satisfaction are estimated. We employ the Actor–Partner Interdependence Model to estimate the effects, using simultaneous equation modeling.

**Results:**

We find significant actor effects of personality, i.e., agreeableness, openness, conscientiousness, and extroversion, but few partner effects on marital satisfaction. In addition to other socioeconomic effects, we find strong actor effects of subjective social status on marital satisfaction for both spouses. The wife’s level of education had a negative effect on her marital satisfaction.

**Discussion:**

The effects of the spouses’ personalities and sociodemographic circumstances on their marital satisfaction fit the Confucian values fostered in China. The wife’s double burden of having a job and taking care of household duties, negatively affecting her marital satisfaction, may be alleviated by proper government policies.

## 1 Introduction

Spouses’ subjective assessments of their marriage quality are influenced by their continuous interactions in daily life, as well as the social and cultural context in which they reside (Karney and Bradbury, 1995). In China two opposing forces converge: a deeply rooted belief and value system based on Confucianism, as well as a Western ideology influenced by China’s openness policy and rapid economic expansion since the 1980s. Su et al. (2015) found that individual attitudes and beliefs regarding marriage tend to be affected by family values in collectivist societies (Dion and Dion, 1993), in contrast to considering marriage as a personal decision based solely on love in individualist societies. Currently, tradition and modernity, and native and foreign influences, are interwoven, affecting people’s subjective perceptions of marital satisfaction (Inglehart and Baker, 2000).

The power and resilience of Confucian heritage are evident in China (Gu, 2011). A fundamental feature of this system is the norm of the male-centered line of descent (Johnson, 1985), shaping conjugal relations between husbands and wives (Su et al., 2023). Traditionally, a husband assumes most household economic responsibilities, while the wife has a complementary role: giving birth and raising a child to facilitate her husband’s productivity (Whyte and Parish, 1985), resulting in women being relegated to a secondary status (Schein, 1997). In addition, both husbands and wives have a joint responsibility to maintain stability, sustainability, and harmony within their marriages. The Confucian view of marital harmony implies that spouses voluntarily sacrifice their own interests to serve their families (Ng, 2019). This view goes beyond Becker’s view (Becker, 1991) on the gender division of market and household labor.

Meanwhile, Western individualistic values challenge China’s traditional marriage and family structure. Liu et al. (2013) show that gender dynamics nowadays is based on economic independence. Couples embracing modern concepts tend to engage in behaviors that promote an equitable relationship, and are less likely to view sharing household responsibilities and decision-making power as a threat to the husband’s identity (Cao et al., 2019a,b). Increased educational attainment among women and their integration into the full-time labor market have altered subjective assessments of marriage and life for both spouses, consequently leading to demographic changes. According to the Ministry of Civil Affairs of China (2017, 2021) the marriage rate in China has declined from 0.99% in 2013 to 0.66% by 2019. Meanwhile, the divorce rate has increased from 0.26% in 2013 to 0.34% in 2019.^1^

Diener et al. (1999) have explored the factors contributing to individual happiness, both within and across countries. Personality traits, demographic and economic variables, explaining marital satisfaction (MS) and happiness among spouses, have often been studied separately (Bian et al., 2015; Otis, 2017). Drivers of MS include personality characteristics (Barelds, 2005; Gattis et al., 2004), age differences (Lee and McKinnish, 2018), and gender differences (Jackson et al., 2014). Furthermore, factors such as gender inequality (Chen et al., 2023), intrahousehold bargaining power (Li, 2023), and subjective social status (Leng et al., 2021) influence happiness of the spouses. To date, however, the continuous interaction of personality and socioeconomic characteristics of the spouses has been neglected in these studies, thus providing little information about the dyadic interaction of the marital happiness factor. Since most of the cited literature is from the West, it is interesting to study whether evidence from China differs from the West. In China, rapid economic and social changes coexist with conflicting forces, such as deeply ingrained gender-specific norms.

The main contribution of our research is the construction and empirical verification of a conceptual model integrating both personality and socioeconomic factors simultaneously using the Actor–Partner Interdependence Model (APIM, Cook and Kenny, 2005), and considering both spouses’ subjective assessments of their MS. It analyzes their combined effects, thus mitigating the possibility of overestimating or underestimating the importance of certain factors (such as personality). Additionally, APIM captures mutual influences among the spouses, which helps to identify the factors related to their individual happiness. At the same time, gender differences in the effects of these factors can be examined, which is important in capturing possible gender inequality in the Chinese context. In the current study, three questions are therefore of interest. What personality traits affect each spouse’s MS in China? What other factors contribute to a happy marriage? And do these effects differ between genders in Chinese families? To answer these questions, we use a large-scale family survey.

### 1.1 Marital satisfaction

MS is one of the most common metrics by which to assess the degree of stability and happiness in a marriage. Throughout most literature, MS has been regarded as the subjective feeling of happiness with a relationship involving continuous interaction of personalities (Tavakol et al., 2017).

We separate the determinants of MS into two categories: structural factors and variable factors (see Table 1). Structural factors include personality, gender, and education, that will remain stable after an individual has chosen a partner (McCrae and Costa, 1982). Therefore, the combination of personalities of the parties is also considered a kind of structure. Most education is acquired before the first marriage (Browning et al., 2014) and will remain stable thereafter. Therefore, education is also considered a structural variable.

**Table 1:** Structural and variable factors.

Structural factors	Personality factors	Big five
	Other factors	Education
Variable factors	Individual factors	Age, health, employment status, social status
	Household factors	Demographic variables, e.g., family size, children ratio
		Economic variables, e.g., assets, liabilities

Variable factors may change throughout the course of a marriage, including factors at both the individual (e.g., employment status) and household level (e.g., family size). As household economic resources, including household net worth and liabilities, are pooled in married couples, they are classified as household level variables.

### 1.2 Conceptual model

**Figure 1:**
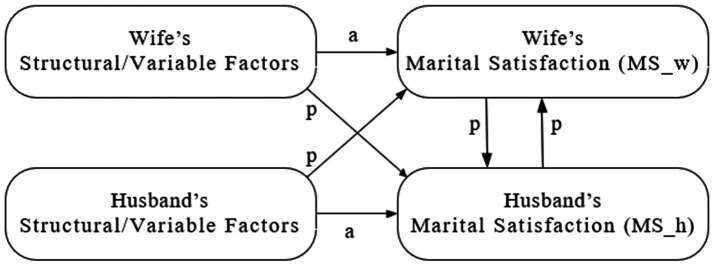
Conceptual model of determinants of marital satisfaction.

A marriage consists of a close dyadic relationship between partners, where one partner’s notions and behaviors indispensably affect the other, which is particularly prevalent in China, where mutual dependence and harmonious relationships are highly valued. Considering the interdependency between spouses, we employ the APIM to construct a conceptual model including the factors affecting happiness. Here, we are interested not only in personality traits but also in socioeconomic factors. In addition, we focus on the reciprocal relationships between wives’ and husbands’ MS (see Figure 1).

APIM is widely used to analyze dyadic data (Cao et al., 2019a,b; Luo et al., 2008). Using APIM, the within- and between-person effects can be investigated simultaneously. Within-person effects, called actor effects (arrows denoted with *a* in Figure 1) represent the effects of one’s own factors on one’s MS. Between-person effects, called partner effects (arrows denoted with *p* in Figure 1), represent the effects of the other spouse’s factors on one’s own MS. All these effects may be either direct, involving only one path, or indirect, involving multiple paths.

### 1.3 Structural factors

Personality traits may affect spouses’ daily communication and experiences, which may significantly impact their satisfaction in a relationship (Donnellan et al., 2004; Dyrenforth et al., 2010). Five-Factor models (Costa and McCrae, 1992) encompass aspects of personality at the highest level of abstraction, including openness to experience, conscientiousness, extroversion, agreeableness, and neuroticism.

Openness refers to the tendency to be creative, imaginative, and intelligent. The actor effect of openness can make individuals perceive marriage life as dull or bored, in turn diminishing their MS (Tsapelas et al., 2009). The partner effect of openness could be explained by a higher propensity to being unfaithful (Orzeck and Lung, 2005) and spending more time exploring various activities and interests, potentially reducing the motivation for maintaining the marital relationship or taking household responsibilities (Solomon and Jackson, 2014), leading to lower levels of the partner’s MS.

Generally, actor effects of extroversion and neuroticism on MS are stronger than partner effects (Barelds, 2005). Extroversion has a positive impact on life satisfaction (Costa and McCrae, 1980) because of its association with being assertive, gregarious, and enthusiastic (Costa and McCrae, 1992). However, neuroticism can adversely impact both spouses’ well-being by negatively interpreting and acting toward life events, and negative interaction with their partners (Sayehmiri et al., 2020; Heller et al., 2004).

Having a high level of conscientiousness is associated with being responsible and self-disciplined (McCrae and Costa, 1991), while being agreeable is associated with cooperation, altruism, and harmonious relationships with others, leading to a higher level of one’s MS. Botwin et al. (1997) found that the husband’s conscientiousness had a positive effect on the wife’s MS, as well as partner effects of agreeableness on both partners’ MS. Claxton et al. (2012) suggests that conscientiousness is the trait most associated with marital satisfaction across all five Big-Five traits, based on a sample of couples who had been married over 20 years.

Similarities in both partners’ personalities are predictors of achieving (Botwin et al., 1997) and maintaining MS (Gonzaga et al., 2010). Assortative mating is significantly related to agreeableness, conscientiousness, and intellect–openness (Botwin et al., 1997). For this reason, we test the effects of personality differences between spouses in the Results section.

In Becker’s model of marriage matching (Becker, 1973, 1974), the level of education is an important factor, particularly at higher levels (Kalmijn, 2013). In addition, evidence shows that men are increasingly considering the economic potential of higher education when selecting a spouse (Oppenheimer et al., 1997). Through sharing the financial burden of modern life with their husbands in the face of increasing costs (Du et al., 2021), women in China with higher education possess greater economic potential and may become more competitive in the marriage market (Peng and Wu, 2022).

Marital satisfaction may be influenced by education due to its effects on expectations and competencies (Tynes, 1990). Education has a significant positive effect on MS in China via both material and spiritual channels (Zhang and Liang, 2023), and on happiness because of self-confidence, self-esteem, employment odds, income, and good health (Cuñado and De Gracia, 2012). Also, women with higher education may attain greater marital equality and improved marital quality in India (Vikram, 2023). However, others find that better-educated women have unrealistically high expectations of marriage, challenging the achievement of a happy marriage (Amato et al., 2003). Furthermore, no relationship between education and MS was found by Malm et al. (2023).

### 1.4 Variable factors

The relationship between age and happiness has been examined within the framework of the family life cycle, showing a U-shaped pattern over time, mostly supported by cross-sectional studies (Orbuch et al., 1996), reaching a minimum in the 35–50 age bracket for happiness. However, based on a fixed-effect pooled time-series model with panel data, VanLaningham et al. (2001) found that MS declined continuously with marital duration in the U.S.

Couples in good physical health are more likely to enjoy their lives, live longer, and are more satisfied with each other’s company. However, mental health problems, such as psychological disorders, may decrease MS (Renne, 1971) by diminished ability to communicate. Carr et al. (2014) found a partner effect of spouses’ self-rated health on the wife’s but not the husband’s life satisfaction.

Employment status in Becker’s theory (Becker, 1991) implies specialization of the partners in housework or market work to gain economically from their marriage based on comparative advantages, thus increasing family welfare. Stutzer and Frey (2006) find evidence for this theory while Oppenheimer (1997) argues that extreme gender specialization might have detrimental effects on the economic security of a family. Davis and Greenstein (2004) found that spouses may not benefit equally from specialization. It is uncertain how employment status affects happiness, as it contributes to women’s financial independence and negotiating power in marriage, which ultimately improves their level of happiness, but also may conflict with their traditional roles as main caregivers. Oshio et al. (2013) show that higher household burdens for women in China lower their marital happiness, since they carry double burdens, both in the labor market and at home.

Generally, subjective social status (SSS), or social rank, is positively correlated with happiness, meaning that people are happier at the top of the social ladder than those at the bottom (Veenhoven, 2016). Subjective measures of social status may differ substantially from objective social status (Evans and Kelley, 2004), the latter typically being indicated by education, income, and occupation. Research found that the explained variance in marital satisfaction in China from SSS is higher than from objective social status (Leng et al., 2021). In Chinese society, SSS is largely determined by one’s social capital (Kim and Lee, 2021) based on a social exchange system called Guanxi, serving as a basis of exchanging favors, resources, and business opportunities (Pearce and Robinson, 2000). Creating relationships with those who have high-status occupations elevates an individual’s SSS by increased access to potential resources, opportunities, and information, thus enhancing emotional and economic benefits for the family (Crombie, 2011).

Correlation between the number of children and a couple’s satisfaction in their marriage depends on how the couple manages their responsibilities, free time, communication, conflicts, finance, etc. Child rearing is mainly expected to be the responsibility of mothers, while fathers are the main financial providers in Confucian culture (Ochiai et al., 2008). In general, having more children may reduce MS (Lee and Ono, 2008; Twenge et al., 2003). According to Glass et al. (2016), in most advanced industrialized countries, parents experience lower happiness than non-parents, whereas in countries with more supportive policies, such as paid time off and child-care subsidies, differences between parents and non-parents are smaller.

Financial distress tends to impact marital quality negatively (Conger et al., 1990). Couples with high economic wealth may participate in activities contributing to stress reduction and conflict resolution (Dakin and Wampler, 2008) resulting in improved marriages. Economic hardship or having debt may increase marital conflict over money and economic pressure, leading to marital dissatisfaction and lower happiness (Dew, 2008; Tay et al., 2017).

### 1.5 Marriage satisfaction and global happiness

Marriage tends to benefit both men and women (e.g., Waite and Lehrer, 2003), although the positive effects of marriage are believed to be stronger for men (Mickelson et al., 2006; Voydanoff and Donnelly, 1999). Marital quality is positively associated with own happiness (i.e., actor effect), contributing more to happiness than any other aspects of life (Glenn and Weaver, 1981; Stutzer and Frey, 2006). In a marriage, the correlation between marital quality and happiness is typically stronger among women than among men, because women generally specialize in nurturing roles, unlike husbands who specialize in employment outside the home (Jackson et al., 2014; Proulx et al., 2007). Gustavson et al. (2016) show that one’s MS is also associated with the spouse’s life satisfaction and well-being (i.e., partner effect). Carr et al. (2014) found evidence of actor effects but no partner effects among elderly couples.

## 2 Methods

### 2.1 Data

This analysis employs the 2018 data from the China Family Panel Studies (CFPS), currently the largest and most comprehensive social survey project in China.^2^ The CFPS baseline sample covers 25 provinces, representing 95% of the Chinese population. In addition, the similarity of the age–sex structure of the CFPS baseline sample and the 2010 Census also indicates the representativeness of the CFPS (Xie and Hu, 2014). Therefore, the CFPS can provide an overall picture regarding MS in China. The CFPS focuses on individuals and families, where both spouses were interviewed. Since personality traits were only collected in the 2018 CFPS wave, we captured a cross-section focusing on the 6,603 couples with a marital status of “married.”

### 2.2 Measures

#### 2.2.1 Marital satisfaction

Chinese culture highlights the role of men as main breadwinners and women as main homemakers. It is important to note that the CFPS data contain dual subjective information, including satisfaction with spousal housework contribution and spousal economic contribution. MS in the CFPS was assessed by three items, as follows: “Are you satisfied with the economic contribution that your spouse/partner makes to the family?,” “Are you satisfied with the contribution on housework that your spouse/partner makes to the family?,” and “In general, are you satisfied with your current marriage?” In Western countries, only the third item is commonly used. However, we have also included the other two items that are very important within the Chinese context. Interviewees indicated their degree of satisfaction to each statement on a 5-point satisfaction scale ranging from 1 (*very dissatisfied*) to 5 (*very satisfied*). The principal component analysis of the items is shown in the Supplementary Table S1, with all three items loading predominantly on one factor (>0.8). Composite reliability (CR) of the three items was 0.868. Convergent validity assessed by the average variance extracted (AVE) was 0.687. Therefore, the average of the three items was used to measure marital satisfaction.

#### 2.2.2 Structural factors

Personality traits were our focal independent variables. In the CFPS 2018, interviewees were asked to rate themselves on a 5-point Likert-type scale running from 1 (*strongly disagree*) to 5 (*strongly agree*), according to three statements for each of the Big-Five personality factors (see Supplementary material). After removing four reversed-scored items (suggested by Wu and Gu, 2020), both CR and AVE for each latent construct were greater than 0.7, indicating sufficient reliability and discriminant validity. Based on the adjusted scale, we calculated the average score of the relevant items within each dimension. In addition, we measured respondents’ educational attainment in terms of their years of education.

#### 2.2.3 Variable factors

We first controlled for a range of demographic characteristics commonly used in MS research. These included individual-level characteristics such as age and self-reported health status, and household level characteristics such as the ratio of dependent children, and family size (excluding dependent children). All variable factors are defined in Table 2.

**Table 2:** Definition of variable factors.

Variable	Definition
Age	Age of the respondent in years
Health	1 if the respondent rates his/her health status as excellent, very good, good, or fair; 0 if he/she rates it as poor
SSS	Average of the respondent’s self-rated relative income level and the respondent’s self-rated social status level in respondent’s local area
Employ	1 if the respondent has a job, 0 otherwise
Children ratio	A family’s number of children under 16 divided by family size
Family size	The number of family members (children under the age of 16 excluded)
Ln net worth	The natural logarithm of the value of “household net worth plus 1” if the net worth is non-negative, the negative value of natural logarithm of the value of “1 minus household net worth” if the net worth is negative^a^
Debt	1 if the family is in debt, 0 otherwise
Rural	1 if the family lives in a rural area, 0 otherwise

a Following Li (2021).

In addition, SSS was included, consisting of the average self-rated relative income level and self-rated social-status level (Zhao et al., 2021) with a correlation coefficient of 0.519. Furthermore, a range of other important economic factors was controlled for, including the couple’s employment status, household net worth, and household debt. Furthermore, we also controlled for the residence (rural or urban) and geographical region (West-, East-, or Central China), to partially capture unobserved socioeconomic and cultural effects.

### 2.3 Estimation strategy

We employed a non-recursive Structural Equation Model, allowing the simultaneous estimation of all relationships in Figure 1. Since the residual errors of the endogenous variables are often correlated, we specified the covariances between the disturbance terms of MS of both wives and husbands (Schaubroeck, 1990). The model was estimated using maximum likelihood and standardized effects were reported.

We included personality traits, educational attainment, physical health, and employment status of both partners into the MS regressions for both wives and husbands. However, the correlation between age of the spouses was too high (0.969) to include these two variables in one regression simultaneously, so we included only the husband’s age in all regressions. To test a U-shaped relationship with age, the squared age was also included.

For the model to be identified, the partner effects of SSS for both spouses had to be restricted to zero (cf. Wong and Law, 1999). Verification of this restriction was obtained by imposing other restrictions and observing the partner effects of SSS. When subsequently partner effects of health and employment were, respectively, restricted to zero, the partner effects of SSS were not significant, thus providing confirmation for our identifying restriction on SSS.

## 3 Results

### 3.1 Sample

Summary statistics for the variables are shown separated by gender in Table 3, indicating significantly higher MS for husbands than for wives. In terms of personality factors, wives showed higher levels of extroversion, agreeableness, and neuroticism, compared to husbands, in line with previous studies (Weisberg et al., 2011), and lower levels of openness, and conscientiousness. In addition, compared to husbands, wives were younger, less educated, less likely to be employed, and less healthy.

**Table 3:** Summary statistics of individual and household characteristics.

	Wife		Husband		
	Mean	SD	Mean	SD	Correlations
**Dependent variable**					
MS	4.216^b^	0.849	4.606 ^b^	0.630	0.251**
**Structural factors**					
Openness	3.062^b^	0.861	3.181^b^	0.867	0.164**
Conscientiousness	3.958^b^	0.667	4.021^b^	0.635	0.142**
Extroversion	3.678^b^	0.868	3.627^b^	0.877	0.063**
Agreeableness	4.074^b^	0.633	4.029^b^	0.625	0.146**
Neuroticism	3.440^b^	0.955	3.107^b^	0.985	0.128**
Education	6.748^b^	4.831	8.387^b^	4.092	0.575**
**Variable factors**					
Age	50.49^b^	12.49	52.35^b^	12.75	0.969**
Health	0.789^b^	0.408	0.853^b^	0.354	0.158**
SSS	3.097^a^	0.945	3.064^a^	0.909	0.248**
Employ	0.726^b^	0.446	0.839^b^	0.367	0.460**
Children ratio	0.094	0.159			
Family size	3.527	1.688			
Ln net worth	11.71	4.271			
Debt	0.342	0.475			
Rural	0.502	0.500			
**Sample characteristics**					
No. of couples	6,603				

The superscripts indicate significant differences between the spouses (^a^*p* < 0.05; ^b^*p* < 0.01); the asterisks indicate significant correlations between the spouses (*p < 0.05; **p < 0.01).

Half of the households in the sample lived in rural areas. Excluding dependent children, the average family size was over three individuals. About 34.2% of households were in debt. The average household net worth was 700,629 RMB (about 105,835 USD at the 2018 price level).

Table 3 further shows the couple correlations for all variables. The couples in our analysis were strongly assorted for age and education. Also, in terms of personality factors, people are likely to attract partners who are similar. Furthermore, the spouses’ MS were positively associated.

### 3.2 Regressions

We conducted two regressions, one including personality traits exclusively (shown in the Supplementary Table S8), and the other including both personality and sociodemographic variables (the full model, shown in Table 4).^3^ The personality coefficients were somewhat lower in the personality-only regression, while the MC^2^ of the full model was higher.^4^ This result shows that the sociodemographic variables contributed significantly to explaining MS of the spouses. Also, the significance of some personality effects was increased, for example, agreeableness and neuroticism, as compared with the personality-only model. Since the model was completely identified, the statistical fit was perfect. The index of stability was 0.339, indicating that the model was stable.

**Table 4:** Estimates from the non-recursive causal model (standardized).

Variables	MS_w	MS_h
	(1)	(2)
MS_w		0.261
		(0.063)**
MS_h	0.443	
	(0.188)*	
Openness_w	0.014^△^	−0.029
	(0.015)	(0.013)*
Openness_h	0.005	−0.036^△^
	(0.015)	(0.014)**
Conscientiousness_w	0.013	0.003^b^
	(0.013)	(0.013)
Conscientiousness_h	0.011	0.048^b^
	(0.018)	(0.014)**
Extroversion_w	0.033^a^	0.000^b^
	(0.013)*	(0.013)
Extroversion_h	−0.006^a^	0.041^b^
	(0.016)	(0.013)**
Agreeableness_w	0.055^a^	0.043^b^
	(0.018)**	(0.014)**
Agreeableness_h	0.003^a^	0.082^b^
	(0.022)	(0.013)**
Neuroticism_w	−0.037	−0.000
	(0.012)**	(0.013)
Neuroticism_h	−0.004	−0.006
	(0.012)	(0.012)
Edu_year_w	−0.089^a△^	0.064^b△△^
	(0.019)**	(0.017)**
Edu_year_h	0.020^a△△^	−0.013^b△^
	(0.015)	(0.015)
Age_h	−0.152	0.017
	(0.092)	(0.093)
Age2_h	0.176	−0.010
	(0.090)*	(0.091)
Health_w	0.024	0.016
	(0.013)	(0.013)
Health_h	0.025	0.037
	(0.016)	(0.013)**
SSS_w	0.179^△^	
	(0.018)**	
SSS_h		0.060^△^
		(0.013)**
Employ_w	−0.028	0.001
	(0.014)*	(0.014)
Employ_h	0.017	0.003
	(0.015)	(0.015)
Children_ratio	−0.040	0.011
	(0.017)*	(0.017)
Familysize	0.004	−0.016
	(0.013)	(0.013)
Ln net worth	−0.000	0.002
	(0.013)	(0.013)
Debt	−0.044^△^	0.002
	(0.013)**	(0.013)
Rural	0.013	−0.018
	(0.013)	(0.013)
Region	Controlled	Controlled
Constant	−0.000	0.000
	(0.012)	(0.012)
No. of couples	6,603	6,603
MC^2^	0.132	0.098

*p < 0.05; **p < 0.01; standard errors in parentheses, the overall *R*^2^ was 0.328.

The superscripts a (b) indicate significant (*p* < 0.05) differences between the actor and the partner effect of each variable on the wife’s (husband’s) MS.

The superscripts △(△△) indicate significant (*p* < 0.05) gender difference in actor (partner) effects of each variable on own MS.

The suffix w (h) indicates the variable of the wife (husband).

Conti and Pudney (2011) show that lower satisfaction scores could be produced if spouses were surveyed together, due to the correlation between spouses’ answers. We checked the robustness of our results by keeping only those being interviewed separately, thus eliminating 2,000 cases from the survey. We obtained roughly similar results as with the full sample.

We conducted two types of coefficient equality tests. The first tested equality of the coefficients of the wife’s characteristics and their husband’s corresponding characteristics within each column. This test was performed only when variables from both partners were included in a single regression. The second test examined if the coefficients of wife’s characteristics and husband’s corresponding characteristics were equal between Columns 1 and 2. The results of these tests are shown in Table 4, using superscript notation for clarity.

#### 3.2.1 Structural factors

We found significant positive actor effects for the wife’s extroversion and agreeableness on their MS, while neuroticism showed a negative effect (see Column 1 in Table 4). The wife’s negative actor effect of neuroticism on her MS could be explained by her tendency of overseeing the marital relationship (Holtzworth-Munroe and Jacobson, 1985), being more sensitive to the unpleasant aspects of the marriage (Thompson and Walker, 1989), and neuroticism amplifying this sensitivity, leading to lower her MS. No partner effects were found between the husband’s personality traits and the wife’s MS. This result confirms prior findings of Barelds (2005) suggesting that marital quality is closely related to the spouses’ own personalities.

Actor effects of the husband’s own conscientiousness, extroversion, and agreeableness were all positively related to their MS, in line with Malouff et al. (2010), while openness showed a negative effect (see Column 2). Obviously, husbands in China are usually regarded as breadwinners. Husbands scoring higher on conscientiousness may tend to work better and earn more, leading to greater norm adherence and higher family status, thus increasing their MS. The positive actor effect of extroversion contributed to the formation of positive and satisfying relationships between spouses. Own agreeableness also showed a strong actor effect on MS across genders. No husband actor effect of neuroticism was found.

Some partner effects for the husband were found. Specifically, the wife’s openness negatively affected the husband’s MS, in line with Solomon and Jackson (2014). The wife’s agreeableness had a positive effect (see Column 2), due to the still dominant expectation for wives to be gentle and obedient. So, with an agreeable wife the husband usually felt more satisfied with his marriage. Additionally, the equality test shows significant differences for conscientiousness, extroversion, and agreeableness between spouses.

The wife’s education had a negative actor effect on her MS, in line with Amato et al. (2003), which may indicate that the traditional gender division of household work conflicts with the women’s increased education levels (Peng and Wu, 2022). The reason for this effect is that a well-educated spouse may have a busy career and the capability of making independent decisions. Surprisingly, a positive partner effect of the wife’s education on the husband’s MS was found. This may be due to the positive impact of a woman’s higher education on the household income. Briefly speaking, a wife’s good education level will improve his MS, but weaken her MS. Also, the equality test shows significant differences for education.

#### 3.2.2 Variable factors

The results showed that MS was hardly affected by age of the spouses. The actor effects of self-reported health on MS were significant for husbands. It might be because men often take on more responsibilities for earning money and doing physical work in the family; then the husband’s masculine dignity may be affected when he is unable to fulfill this role.

SSS consistently showed strong actor effects for both spouses on MS, albeit for different reasons (see Section 3.2.3). In particular, the coefficient of wives’ SSS was 0.179, which ranks second among all the factors that affected own MS, as compared to the coefficient of husbands’ MS (0.443) on wives’ MS. A high SSS usually is associated with relatively high income, as well as individual reputation, trust, and reciprocity (Crombie, 2011), resulting in better living conditions and more attractive environments than those with lower SSS. Meanwhile, higher SSS is closely related to Guanxi in the Chinese context, which is instrumental in obtaining economic benefits, information, and resources both in the present and in the future.

The wife’s employment status had a significant negative actor effect. In China, full-time jobs are more common than in other Asian countries. The percentage of working wives under 55 years of age in our sample was 83%. To some extent a wife’s employment deviates from gender norms in marriage, which may lead to dissatisfaction (Chen and Hu, 2021). Her combined role as a caregiver for her family and as a paid worker can increase her workload, undermining her happiness (Yu and Liu, 2021). Unexpectedly, there were no actor or partner effects of the husbands’ employment status on both partners’ MS. This can be attributed to the fact that in our sample only 16% of husbands were unemployed, and this subgroup had an average age of 64.8, indicating that most of them were retired.

Children ratio had a significant negative impact on the wife’s MS, in accordance with other East Asian societies with Confucian culture, because wives often bear the childrearing burden of dependent children, and institutional support for childcare tend to be weak in these societies.

We can see that the MS of one’s spouse was positively related to one’s own MS, implying that the spillover effect of MS from one partner to the other was strong. Having a marriage requires daily coordination and interaction regarding a variety of issues. This practice increases compatibility and reduces conflict, the absence of it may negatively impact marital satisfaction. It is not surprising that women’s MS depends largely on their husbands’ MS, in line with Confucian hierarchical norms, that is, wives are subordinate to their husbands.

#### 3.2.3 Gender differences

The deltas in Table 4 represent significant intra-household actor/partner-effect differences. The results showed that intra-household gender differences were found not only in the actor effects of openness, SSS, and debt, but also in the actor and partner effects of education.

One such difference is between spouses’ SSS actor effects on their MS. The results suggest that higher self-perceived social status contributes to greater MS for wives than for husbands. Traditionally, in Chinese society, the husband is more educated and earns more than his wife, and the family’s social relationships are also more dependent on men, which is reflected in a well-known Chinese proverb: “Men handle external affairs, while women take care of domestic matters.” In such cases, the wife may be more satisfied with the marriage due to “marrying up,” thus increasing her happiness in life.

Financial security is a source of family happiness (Schaninger and Buss, 1986). However, the impact of household debts on spouses’ happiness varies significantly by gender. Specifically, debts have no significant impact on the husband’s MS but significantly undermine the wife’s MS.

#### 3.2.4 Other effects

Direct and indirect effects are added up to determine the total effect of an independent variable on the dependent variable. For example, the standardized direct effect of a wife’s agreeableness on her own MS equaled 0.055 (see Table 4). The sum of the standardized indirect effects of her agreeableness on her MS equaled 0.029 (see the Supplementary Table S10). Therefore, the standardized total effect equaled 0.084 (see the Supplementary Table S10). This means that her agreeableness continues to have the greatest direct impact on MS.

## 4 Discussion

Using dyadic data in the 2018 wave of the China Family Panel Studies, combining personality and socioeconomic factors, our research illustrates a complex picture of the actor and partner relationships between wives’ and husbands’ MS highlighting the factors associated with marital satisfaction in the Chinese cultural context. In our study, sociodemographic factors contributed significantly to explaining MS of the spouses and their inclusion increased the significance of some personality effects implying that the exclusion of sociodemographic variables in previous studies has led to underestimation of the personality effects.

Marital quality is associated with both partners’ own personality traits (Robins et al., 2000). In Chinese families, agreeableness shows stronger actor effects on MS, for both men and women, than the other traits. Apparently, actor effects of extroversion and agreeableness were observed on both spouses’ MS, implying that their own personalities were associated with these life outcomes. Furthermore, considering the stability of personality, selecting a partner with a certain personality trait seems to be relatively important. For the husband, having an agreeable wife is an important factor to improve his marital happiness, according to our findings. However, we did not find evidence of the influence of an agreeable husband on his wife’s marital satisfaction. This is because the traditional gender norm for women is to be obedient, which is a commendable female image, that is, she is willing to give priority to her husband and family. In addition, Ying (1991) stated that Chinese marital quality is not influenced by emotional interaction within the marriage, showing that, traditionally, good matches and successful marriages in Chinese culture were not always dependent on compatibility and romantic interest. Also, we did not find a positive effect of the husband’s conscientiousness on the wife’s MS, possibly because this trait is often related to rationality and discipline.

It is important to note that the actor effects of SSS on MS were significant, for both spouses. Our measurement of SSS encompasses subjective appraisals of personal income and societal standing, intricately intertwined with culturally specific factors such as interpersonal relationships and individual achievements within the framework of Chinese values. In the Chinese cultural context, SSS values social relationships and connectedness in addition to being a symbol of personal achievement and success, significantly influencing happiness. Historically, Chinese scholars were eager to excel in imperial examinations, the primary avenue for individuals from humble backgrounds to improve their SSS (Wang et al., 2014). Even in modern times, Chinese children often carry the hopes of an entire family, aspiring them to attain higher SSS as adults. In China, resources—developed by Guanxi—are closely tied to SSS. Individuals or families with higher SSS enjoy greater access to political, educational, business, and healthcare resources. These resources, in turn, help them to overcome challenges more easily at work or in life for themselves and their family members, leading to happiness, which differs from Western countries, as well as other East-Asian countries with Confucian cultural background.

Gender roles in the Chinese cultural context can be understood through three themes: labor role differentiation between spouses, marriage and career importance for women, and giving birth to children (Cao et al., 2019a,b). Women’s education and full-time work are related to these three themes.

Regarding education, we find that the wife’s higher education level can weaken her MS but contributes to her husband’s MS. This finding shows that Chinese wives have become increasingly important sources of household income, which differs from other East-Asian countries. In contrast, considering their limited energy and time, women must strike a balance between career development and household duties. On one hand, considering the increasing convergence between men’s and women’s educational attainment, highly educated women are increasingly excluded from the marriage market (Qian and Qian, 2014). On the other hand, higher education may result in men performing household chores for their families, contrary to the traditional division of labor between men and women. Therefore, it is understandable that higher education can negatively affect the wife’s MS. According to Bittman et al. (2003), women tend to compensate for their higher earnings by performing more housework. Chen and Hu (2021) point to increasing intrahousehold problems in China when the wife earns more than the husband.

Employment and children ratios tends to negatively affect the wife’s MS, but not the husband’s MS. Our finding shows that wives in China often find themselves trapped between traditionalism and modernism, the former expecting them to take more domestic responsibilities, while the latter requires them to be independent workers, involving certain sacrifices. Women’s earnings are an important part of most family budgets but the association with stress from full-time work, and childcare tends to undermine the wife’s MS (see Table 4). This finding is consistent with the agreement of Chinese respondents with the statement in the World Values Survey of “Being a housewife is just as fulfilling as working for pay,” which changed from 60% in 2013 to 71.6% in 2018. This fact provides further evidence for Chinese gender norms becoming more traditional over time, thus confirming that traditional gender values remain deeply ingrained (Gui, 2020).

In China, women often face the dilemma between maintaining family harmony and participating in economic activities—a challenge not typically encountered by men. This dilemma reflects the persistent gender inequality within Chinese households. Apparently, the demands of full-time work and childcare, i.e., double burden, lower the wife’s marital happiness. Rooted in Confucian culture, values such as order, conformity, and collective interests are emphasized (Diener and Diener, 1995; Uchida et al., 2004), underpinning traditional, male-dominant role patterns, where men are primarily responsible for earning income while women take on caregiving roles. This division of labor contributes to gender disparities in well-being, which tend to favor men (Hori and Kamo, 2018). This process may explain to some extent why young people in China are afraid of marriage and childbirth. Policies provided by the government, including affordable rates of childcare service, insurance of birth-related career breaks, and advocating husbands’ contribution in childcare to the family may be desirable in China (Yu et al., 2019). In addition, our observations indicate that 64% of households borrow money for housing, which may be the main cause of financial pressure. Thus, the government may promote financial literacy education among the public, possibly alleviating household debt burdens, and reducing the likelihood of residents experiencing debt hardships, thereby improving the quality of life for families.

### 4.1 Limitations

This study has some limitations. Some selection bias may exist since the sample excludes individuals who divorced due to extreme dissatisfaction with their marriage. Although divorce rates are relatively low in China, by studying how structural factors affect divorce, the whole picture may become more coherent. In addition, due to the cross-sectional nature of the data collected, the statistical relationships in this analysis may not be considered as causal. To gain insight into causal relationships, future studies may consider longitudinal data.

## Data Availability

Publicly available datasets were analyzed in this study. This data can be found here: https://www.isss.pku.edu.cn/cfps/en/.

## Footnotes

^1^Marriage (divorce) rate = (Number of marriages(divorces) in the current year/average total population in the current year)*100%. These figures exclude the COVID period.

^2^CFPS data is available at https://www.isss.pku.edu.cn/cfps/en/.

^3^In the personality-only regression, we applied restrictions for identification on the coefficients that were almost equal to zero in the fully specified model. The unstandardized coefficients from the full model are shown in the Supplementary Table S9.

^4^MC^2^ is recommended by Bentler and Raykov (2000) to be used instead of R-squared for non-recursive systems.
