# Supplementary material for: Spouses’ personalities and marital satisfaction in Chinese families
Source: Front Psychol. 2025 Feb 5;16:1480570. doi: 10.3389/fpsyg.2025.1480570 (PMC11835996; doi:10.3389/fpsyg.2025.1480570)
Supplement: Supplementary file 1 [file Table_1.docx]

**Spouses’ Personalities and Marital Satisfaction in Chinese Families**

**Supplementary Material**

This supplement provides detailed information about the sample, the scales used in the analysis, and the results. Tab s1 shows summary statistics of the relevant scales.

In Table S2 the factor loadings of the MS items indicate unidimensional scales. Since the factor loadings were roughly equal, we used the average of the scale items in the analysis.

As Table S3 shows, the original 15-item Big Five scale includes 11 positively worded items and four negatively worded items. Principal component analysis with varimax rotation was used to investigate the factor structure of the original scale. The results are reported in Tab s4, showing that the three reversed-scored items for the extroversion, agreeableness, and neuroticism dimensions were the poorer-loading items on the corresponding components, which is consistent with findings by Carciofo et al. (2016).

Next, we evaluated the Big Five scale by analyzing internal consistency. As shown in the first column of Table S5, Cronbach’s alphas of the four dimensions including reverse-scored items were all below 0.4. This is because the positively worded items are likely to be statistically and practically different from the negatively worded items within each dimension (Weems & Onwuegbuzie, 2001). Meanwhile, we found that the consistency of each personality trait was maximized after removing the reverse stated question. Therefore, combining the results of principal component analysis with the results of the internal consistency estimates, we decided to retain the 11 positively worded items. Cronbach’s alphas of the adjusted scale are shown in the second column of Table S5. Although somewhat inferior to some multi-item scales such as BFI-44 (Carciofo et al., 2016), Cronbach’s alphas of the adjusted scale reached similar levels as those of other brief measure of the Big Five such as the GSOEP scale and the TIPI (Gosling et al., 2003; Hahn et al., 2012), shown in the last two columns of Tab s6.

The adjusted scale reliability was further measured by calculating composite reliability coefficients (CR) and the convergent validity by average variance extracted (AVE). Table S6 shows that the CR values of all dimensions were higher than 0.7, a rule-of-thumb critical value, once again indicating that the adjusted scale has reasonable internal consistency. The AVE values of openness, conscientiousness, extroversion, agreeableness, and neuroticism were 0.518, 0.567, 0.636, 0.602, 0.667 respectively, all exceeding the threshold of 0.5 and verifying convergent validity. Furthermore, we could see from Table S6 that the discriminant validity was verified because the values on the diagonal are the highest in any column or row (except the composite reliability) (Fornell & Larcker, 1981).

Finally, Table S7 shows the unidimensional structure of the SSS scale.

**Table S1** Summary statistics of individual and household characteristics.

|  | Wives | | | |  | Husbands | | | |  | Correlations |
| --- | --- | --- | --- | --- | --- | --- | --- | --- | --- | --- | --- |
|  | Mean | SD | Min | Max |  | Mean | SD | Min | Max |  |  |
| MS | 4.216^a^ | 0.849 | 1 | 5 |  | 4.606^a^ | 0.630 | 1 | 5 |  | 0.251** |
| *Structural factors* |  |  |  |  |  |  |  |  |  |  |  |
| Openness | 3.062^a^ | 0.861 | 1 | 5 |  | 3.181^a^ | 0.867 | 1 | 5 |  | 0.164** |
| Conscientiousness | 3.958^a^ | 0.667 | 1 | 5 |  | 4.021^a^ | 0.635 | 1 | 5 |  | 0.142** |
| Extroversion | 3.678^a^ | 0.868 | 1 | 5 |  | 3.627^a^ | 0.877 | 1 | 5 |  | 0.063** |
| Agreeableness | 4.074^a^ | 0.633 | 1 | 5 |  | 4.029^a^ | 0.625 | 1 | 5 |  | 0.146** |
| Neuroticism | 3.440^a^ | 0.955 | 1 | 5 |  | 3.107^a^ | 0.985 | 1 | 5 |  | 0.128** |
| Edu year | 6.748^a^ | 4.831 | 0 | 19 |  | 8.387^a^ | 4.092 | 0 | 22 |  | 0.575** |
| *Variable factors* |  |  |  |  |  |  |  |  |  |  |  |
| Age | 50.49^a^ | 12.49 | 20 | 86 |  | 52.35^a^ | 12.75 | 22 | 93 |  | 0.969** |
| Health | 0.789^a^ | 0.408 | 0 | 1 |  | 0.853^a^ | 0.354 | 0 | 1 |  | 0.158** |
| SSS | 3.097^b^ | 0.945 | 1 | 5 |  | 3.064^b^ | 0.909 | 1 | 5 |  | 0.248** |
| Employ | 0.726^a^ | 0.446 | 0 | 1 |  | 0.839^a^ | 0.367 | 0 | 1 |  | 0.460** |
| Children ratio | 0.094 | 0.159 | 0 | 0.714 |  |  |  |  |  |  |  |
| Family size | 3.527 | 1.688 | 2 | 16 |  |  |  |  |  |  |  |
| Ln net worth | 11.71 | 4.271 | -14.05 | 17.73 |  |  |  |  |  |  |  |
| Debt | 0.342 | 0.475 | 0 | 1 |  |  |  |  |  |  |  |
| Rural | 0.502 | 0.500 | 0 | 1 |  |  |  |  |  |  |  |
| *Sample characteristics* |  |  |  |  |  |  |  |  |  |  |  |
| No. of couples | 6,603 |  |  |  |  |  |  |  |  |  |  |

**Table S2** Factor structure of MS

| Items | MS |
| --- | --- |
| Degree of satisfaction toward current marriage/cohabitation | 0.835 |
| Degree of satisfaction toward your partner's economic contribution to your family | 0.847 |
| Degree of satisfaction toward your partner's housework contribution | 0.804 |

**Table S3** Original Big Five scale in CFPS2018

| Dimensions | CFPS statements |
| --- | --- |
| Openness | (O1): I am original and come up with new ideas. |
|  | (O2): I value artistic experiences. |
|  | (O3): I have an active imagination. |
| Conscientiousness | (C1): I do a thorough job. |
|  | (C2): I do things efficiently. |
|  | (C3R): I tend to be lazy. |
| Extroversion | (E1): I am talkative. |
|  | (E2): I am outgoing and sociable. |
|  | (E3R): I am reserved. |
| Agreeableness | (A1): I have a forgiving nature. |
|  | (A2): I am considerate and kind to almost everyone. |
|  | (A3R): I am sometimes rude to others. |
| Neuroticism | (N1): I worry a lot. |
|  | (N2): I get nervous easily. |
|  | (N3R): I am relaxed and handle stress well. |

Note: [R] = Reverse-scored items (adjusted such that all item loadings for a personality dimension would be positive).

**Table S4** Factor structure of the Big Five personality traits

| Items | Openness | Conscientiousness | Extroversion | Agreeableness | Neuroticism |
| --- | --- | --- | --- | --- | --- |
| O1 | **0.722** | 0.028 | 0.185 | -0.050 | 0.091 |
| O2 | **0.696** | 0.020 | 0.051 | 0.075 | 0.036 |
| O3 | **0.696** | 0.014 | 0.066 | 0.230 | 0.046 |
| C1 | 0.175 | **0.543** | 0.149 | 0.272 | 0.130 |
| C2 | **0.404** | **0.416** | 0.090 | 0.295 | 0.125 |
| C3R | -0.085 | **0.805** | -0.042 | -0.047 | -0.156 |
| E1 | 0.171 | 0.060 | **0.721** | 0.194 | 0.044 |
| E2 | 0.231 | 0.105 | **0.578** | 0.384 | -0.040 |
| E3R | -0.232 | 0.143 | **0.448** | -0.308 | -0.422 |
| A1 | 0.014 | 0.092 | 0.139 | **0.718** | 0.069 |
| A2 | 0.075 | 0.185 | 0.023 | **0.707** | 0.131 |
| A3R | -0.143 | 0.362 | -0.481 | **0.161** | -0.306 |
| N1 | 0.036 | 0.103 | 0.129 | 0.030 | **0.766** |
| N2 | 0.065 | -0.112 | -0.044 | 0.030 | **0.757** |
| N3R | -0.347 | 0.084 | -0.070 | -0.545 | **0.235** |

Note: All loadings >0.4 are in bold type.

**Table S5** Cronbach’s alphas of the CFPS, GSOEP Big Five Scale and the TIPI

| Dimensions | CFPS original scale | CFPS adjusted scale | GSOEP scale | TIPI scale |
| --- | --- | --- | --- | --- |
| Openness | 0.603 | 0.603 | 0.580 | 0.450 |
| Conscientiousness | 0.388 | 0.450 | 0.600 | 0.500 |
| Extroversion | 0.321 | 0.562 | 0.760 | 0.680 |
| Agreeableness | 0.272 | 0.518 | 0.440 | 0.400 |
| Neuroticism | 0.345 | 0.520 | 0.660 | 0.730 |

**Table S6** Reliability and discriminant validity of personality trait measures

|  | O | C | E | A | N |
| --- | --- | --- | --- | --- | --- |
| Openness [O] | 0.72 |  |  |  |  |
| Conscientiousness [C] | 0.328 | 0.753 |  |  |  |
| Extroversion [E] | 0.359 | 0.310 | 0.797 |  |  |
| Agreeableness [A] | 0.262 | 0.376 | 0.308 | 0.776 |  |
| Neuroticism [N] | 0.165 | 0.098 | 0.071 | 0.126 | 0.817 |
| Composite reliability (CR) | 0.763 | 0.718 | 0.776 | 0.752 | 0.800 |

Note: The diagonal reports the square root of the average variance extracted (AVE) for each latent construct, which indicates discriminant validity because the diagonal values are the highest in any column or row (except the CR).

**Table S7** Factor structure of SSS

| Items | SSS |
| --- | --- |
| Your relative income level in your local area | 0.871 |
| Your social status level in your local area | 0.871 |

**Table S8** Non-recursive model with personality factors only (standardized)

|  | (1) | (2) |
| --- | --- | --- |
| VARIABLES | MS_w | MS_h |
|  |  |  |
| MS_w |  | 0.532 |
|  |  | (0.202)** |
| MS_h | 0.799 |  |
|  | (0.617) |  |
| Openness_w | 0.041 | -0.032 |
|  | (0.019)* | (0.014)* |
| Openness_h | 0.008 | -0.019 |
|  | (0.024) | (0.015) |
| Conscientiousness_w | 0.030 | -0.009 |
|  | (0.017) | (0.016) |
| Conscientiousness_h | -0.006 | 0.039 |
|  | (0.042) | (0.017)* |
| Extroversion_w | 0.046 | -0.015 |
|  | (0.018)* | (0.018) |
| Extroversion_h | -0.003 | 0.031 |
|  | (0.035) | (0.016) |
| Agreeableness_w | 0.033 | 0.025 |
|  | (0.048) | (0.022) |
| Agreeableness_h | -0.029 | 0.071 |
|  | (0.061) | (0.017)** |
| Neuroticism_w | -0.036 |  |
|  | (0.026) |  |
| Neuroticism_h |  | -0.013 |
|  |  | (0.014) |
| Constant | -0.000 | 0.000 |
|  | (0.014) | (0.012) |
|  |  |  |
| Observations | 6,603 | 6,603 |
| MC^2^ | 0.070 | 0.075 |

Note: * *p*<0.05, ** *p*<0.01; Standard errors in parentheses

**Table S9** Estimates from the non-recursive causal model (non-standardized)

|  | (1) | (2) |
| --- | --- | --- |
| VARIABLES | MS_w | MS_h |
|  |  |  |
| MS_w |  | 0.194 |
|  |  | (0.046)** |
| MS_h | 0.596 |  |
|  | (0.253)* |  |
| Openness_w | 0.014^△^ | -0.021 |
|  | (0.014) | (0.010)* |
| Openness_h | 0.005 | -0.026^△^ |
|  | (0.015) | (0.010)** |
| Conscientiousness_w | 0.016 | 0.003 *^b^* |
|  | (0.017) | (0.013) |
| Conscientiousness_h | 0.014 | 0.048 *^b^* |
|  | (0.024) | (0.014)** |
| Extroversion_w | 0.032 *^a^* | 0.000 *^b^* |
|  | (0.013)* | (0.010) |
| Extroversion_h | -0.006 *^a^* | 0.029 *^b^* |
|  | (0.016) | (0.010)** |
| Agreeableness_w | 0.074 *^a^* | 0.043 *^b^* |
|  | (0.024)** | (0.014)** |
| Agreeableness_h | 0.004 *^a^* | 0.083 *^b^* |
|  | (0.030) | (0.014)** |
| Neuroticism_w | -0.033^△^ | -0.000 |
|  | (0.011)** | (0.008) |
| Neuroticism_h | -0.003 | -0.004^△^ |
|  | (0.011) | (0.008) |
| Edu_year_w | -0.016 *^a^*^△^ | 0.008 *^b^* |
|  | (0.003)** | (0.002)** |
| Edu_year_h | 0.004 *^a^* | -0.002 *^b^*^△^ |
|  | (0.003) | (0.002) |
| Age_h | -0.010 | 0.001 |
|  | (0.006) | (0.005) |
| Age2_h | 0.011 | -0.000 |
|  | (0.006)* | (0.004) |
| Health_w | 0.049 | 0.025 |
|  | (0.028) | (0.020) |
| Health_h | 0.060 | 0.066 |
|  | (0.039) | (0.023)** |
| SSS_w | 0.161^△^ |  |
|  | (0.016)** |  |
| SSS_h |  | 0.041^△^ |
|  |  | (0.009)** |
| Employ_w | -0.054 | 0.001 |
|  | (0.026)* | (0.019) |
| Employ_h | 0.040 | 0.005 |
|  | (0.035) | (0.026) |
| Children_ratio | -0.212 | 0.043 |
|  | (0.089)* | (0.067) |
| Familysize | 0.002 | -0.006 |
|  | (0.007) | (0.005) |
| Ln net worth | -0.000 | 0.000 |
|  | (0.002) | (0.002) |
| Debt | -0.078^△^ | 0.002^△^ |
|  | (0.023)** | (0.017) |
| Rural | 0.022 | -0.022 |
|  | (0.023) | (0.017) |
| Region | Controlled | Controlled |
| Constant | 0.750 | 2.903 |
|  | (0.903) | (0.206)** |
| Observations | 6,603 | 6,603 |
| MC^2^ | 0.132 | 0.098 |

Notes:

1. * *p*<.05; ** *p*<.01; Standard errors in parentheses; the overall R^2^ was 0.328;
2. The superscripts *a* (*b* ) indicate significant (*p*<.05) differences between the effects of wife’s and husband’s reports of each variable on the wife’s (husband’s) MS;
3. The superscripts △ indicates significant (*p*<.05) gender difference on the diagonal between the effects of a wife’s and husband’s reports of each variable on own MS.

**Table S10** Indirect effects and total effects (standardized)

|  | Indirect_effects | | Total_effects | |
| --- | --- | --- | --- | --- |
|  | MS_w | MS_h | MS_w | MS_h |
| MS_w | 0.131 | 0.034 | 0.131 | 0.295** |
|  | (0.075) | (0.025) | (0.075) | (0.083) |
| MS_h | 0.058 | 0.131 | 0.500* | 0.131 |
|  | (0.056) | (0.075) | (0.242) | (0.075) |
| Openness_w | -0.013 | 0.000 | 0.001 | -0.029* |
|  | (0.008) | (0.003) | (0.013) | (0.014) |
| Openness_h | -0.018 | -0.003 | -0.013 | -0.040** |
|  | (0.010) | (0.004) | (0.014) | (0.014) |
| Conscientiousness_w | 0.003 | 0.004 | 0.016 | 0.007 |
|  | (0.006) | (0.004) | (0.013) | (0.014) |
| Conscientiousness_h | 0.025^*^ | 0.009^*^ | 0.036** | 0.057** |
|  | (0.012) | (0.004) | (0.013) | (0.014) |
| Extroversion_w | 0.005 | 0.010^*^ | 0.037** | 0.010 |
|  | (0.006) | (0.004) | (0.013) | (0.013) |
| Extroversion_h | 0.020 | 0.004 | 0.013 | 0.044** |
|  | (0.010) | (0.004) | (0.013) | (0.014) |
| Agreeableness_w | 0.029^*^ | 0.022^***^ | 0.084** | 0.065** |
|  | (0.014) | (0.006) | (0.013) | (0.013) |
| Agreeableness_h | 0.042^*^ | 0.012^**^ | 0.044** | 0.094** |
|  | (0.019) | (0.004) | (0.013) | (0.014) |
| Neuroticism_w | -0.005 | -0.011^**^ | -0.042** | -0.011 |
|  | (0.006) | (0.004) | (0.012) | (0.013) |
| Neuroticism_h | -0.003 | -0.002 | -0.007 | -0.007 |
|  | (0.006) | (0.003) | (0.012) | (0.013) |
| Edu_year_w | 0.020 | -0.018^**^ | -0.069** | 0.046** |
|  | (0.011) | (0.006) | (0.016) | (0.017) |
| Edu_year_h | -0.004 | 0.004 | 0.016 | -0.009 |
|  | (0.007) | (0.004) | (0.015) | (0.015) |
| Age_h | -0.011 | -0.043 | -0.163 | -0.026 |
|  | (0.042) | (0.026) | (0.091) | (0.094) |
| Age2_h | 0.018 | 0.051 | 0.195* | 0.041 |
|  | (0.041) | (0.026) | (0.089) | (0.092) |
| Health_w | 0.011 | 0.009^*^ | 0.035** | 0.026* |
|  | (0.007) | (0.004) | (0.013) | (0.013) |
| Health_h | 0.022^*^ | 0.012^**^ | 0.047** | 0.049** |
|  | (0.011) | (0.004) | (0.012) | (0.013) |
| SSS_w | 0.023^*^ | 0.053^***^ | 0.203** | 0.053** |
|  | (0.012) | (0.013) | (0.013) | (0.013) |
| SSS_h | 0.030^*^ | 0.008^*^ | 0.030* | 0.067** |
|  | (0.013) | (0.004) | (0.013) | (0.013) |
| Employ_w | -0.003 | -0.008^*^ | -0.032* | -0.007 |
|  | (0.006) | (0.004) | (0.014) | (0.014) |
| Employ_h | 0.004 | 0.005 | 0.021 | 0.008 |
|  | (0.007) | (0.004) | (0.015) | (0.015) |
| Children_ratio | 0.000 | -0.010^*^ | -0.039* | 0.001 |
|  | (0.008) | (0.005) | (0.017) | (0.017) |
| Familysize | -0.007 | -0.001 | -0.003 | -0.016 |
|  | (0.007) | (0.003) | (0.013) | (0.013) |
| Ln net worth | 0.001 | 0.000 | 0.001 | 0.003 |
|  | (0.006) | (0.003) | (0.012) | (0.013) |
| Debt | -0.005 | -0.013^**^ | -0.048** | -0.011 |
|  | (0.006) | (0.004) | (0.013) | (0.013) |
| Rural | -0.007 | 0.002 | 0.006 | -0.016 |
|  | (0.007) | (0.003) | (0.013) | (0.014) |
| West | -0.023^*^ | -0.008^*^ | -0.032* | -0.053** |
|  | (0.012) | (0.004) | (0.013) | (0.014) |
| Central | -0.018 | -0.014^**^ | -0.054** | -0.042** |
|  | (0.010) | (0.005) | (0.013) | (0.013) |
| *N* | 6603 |  | 6603 |  |

Note: * *p*<.05; ** *p*<.01; Standard errors in parentheses.

**References**

Carciofo, R., Yang, J., Song, N., Du, F., & Zhang, K. (2016). Psychometric evaluation of Chinese-language 44-item and 10-item big five personality inventories, including correlations with chronotype, mindfulness and mind wandering. *PloS One*, *11*(2), e0149963. https://doi.org/10.1371/journal.pone.0149963

Fornell, C., & Larcker, D. F. (1981). Evaluating structural equation models with unobservable variables and measurement error. *Journal of Marketing Research*, *18*(1), 39-50. https://doi.org/10.1177/002224378101800104

Gosling, S. D., Rentfrow, P. J., & Swann Jr, W. B. (2003). A very brief measure of the Big-Five personality domains. *Journal of Research in Personality*, *37*(6), 504-528. https://doi.org/10.1016/S0092-6566(03)00046-1

Weems, G. H., & Onwuegbuzie, A. J. (2001). The impact of midpoint responses and reverse coding on survey data. *Measurement and Evaluation in Counseling and Development*, *34*(3), 166-176. https://doi.org/10.1080/07481756.2002.12069033
